# Identification and Validation of Genetic Variants that Influence Transcription Factor and Cell Signaling Protein Levels

**DOI:** 10.1016/j.ajhg.2014.07.005

**Published:** 2014-08-07

**Authors:** Ronald J. Hause, Amy L. Stark, Nirav N. Antao, Lidija K. Gorsic, Sophie H. Chung, Christopher D. Brown, Shan S. Wong, Daniel F. Gill, Jamie L. Myers, Lida Anita To, Kevin P. White, M. Eileen Dolan, Richard Baker Jones

**Affiliations:** 1 Institute for Genomics and Systems Biology, University of Chicago, Chicago, IL 60637, USA; 2 Committee on Genetics, Genomics and Systems Biology, University of Chicago, Chicago, IL 60637, USA; 3 Ben May Department for Cancer Research, University of Chicago, Chicago, IL 60637, USA; 4 Department of Medicine, University of Chicago, Chicago, IL 60637, USA; 5 Department of Genetics, Perelman School of Medicine, University of Pennsylvania, Philadelphia, PA 19104, USA; 6 Department of Human Genetics, University of Chicago, Chicago, IL 60637, USA; 7 Committee on Clinical Pharmacology and Pharmacogenomics, University of Chicago, Chicago, IL 60637, USA

## Abstract

Many genetic variants associated with human disease have been found to be associated with alterations in mRNA expression. Although it is commonly assumed that mRNA expression changes will lead to consequent changes in protein levels, methodological challenges have limited our ability to test the degree to which this assumption holds true. Here, we further developed the micro-western array approach and globally examined relationships between human genetic variation and cellular protein levels. We collected more than 250,000 protein level measurements comprising 441 transcription factor and signaling protein isoforms across 68 Yoruba (YRI) HapMap lymphoblastoid cell lines (LCLs) and identified 12 *cis* and 160 *trans* protein level QTLs (pQTLs) at a false discovery rate (FDR) of 20%. Whereas up to two thirds of *cis* mRNA expression QTLs (eQTLs) were also pQTLs, many pQTLs were not associated with mRNA expression. Notably, we replicated and functionally validated a *trans* pQTL relationship between the KARS lysyl-tRNA synthetase locus and levels of the DIDO1 protein. This study demonstrates proof of concept in applying an antibody-based microarray approach to iteratively measure the levels of human proteins and relate these levels to human genome variation and other genomic data sets. Our results suggest that protein-based mechanisms might functionally buffer genetic alterations that influence mRNA expression levels and that pQTLs might contribute phenotypic diversity to a human population independently of influences on mRNA expression.

## Introduction

Our ability to sequence genomes at an ever-increasing rate has resulted in the identification of many new common and rare genetic variants across human populations.^1–3^ Much effort has been devoted to identifying relationships between genetic variation and complex human phenotypes, including susceptibility to disease and adverse drug response.^4–6^ Developing a mechanistic biological understanding of such statistical associations represents a major ongoing challenge in human genomics.

Expression quantitative trait locus (eQTL) mapping has been used to identify gene targets and mechanisms that link genome variation with complex phenotypic traits.^7–9^ A fundamental assumption made in such studies is that genome variants associated with mRNA expression variation will also be associated with protein-level variation that impacts a trait. Although the influence of genetic variation on mRNA levels may extend to protein levels, many posttranscriptional mechanisms, such as mRNA translation efficiency, protein stability and function, and posttranslational modification, can buffer changes in mRNA expression. Moreover, these same mechanisms can introduce changes in protein levels under conditions of invariant mRNA expression. Such protein-centric mechanisms can be deciphered only by measuring genetic-, mRNA-, and protein-level variation among a population of individuals. Indeed, previous examinations of genetic influences on protein-level variation have observed markedly nonoverlapping loci regulating protein and transcript levels.^10–12^

Unfortunately, we have been unable to globally compare mRNA and protein levels with genetic variation across human populations primarily because of the nonoverlapping gene sets typically collected with current mRNA and protein analysis platforms. Although mass spectrometers (MSs) and MS-based protein analysis methods continue to improve and can quantify thousands of proteins per sample, they currently lack the sensitivity required to consistently observe more than a fraction of the human proteome without depleting highly abundant proteins.^13^ A major problem for most population-level proteome-by-transcriptome comparisons employing mass spectrometry is the biased sampling of proteins across samples; typically, subsets of proteins are detected and quantified in some samples but undetected in others.^10,11,14,15^ This biased detection issue coupled with bias to observe and quantify the most abundant proteins within a sample^16^ results in reduced power to assess the relative contributions of genome influences to the proteome. To better relate genomes to transcriptomes and proteomes, we and others have developed and applied complementary antibody-based “protein-omic” approaches to more reproducibly quantify targeted sets of protein families across individuals provided the availability of validated antibodies directed against the proteins of interest.^17^ We previously coined the term protein-omic to refer to studies that collect information on targeted subsets of functionally related proteins, by contrast to proteomic that refers to larger, more random sampling-based analyses of the proteome, typically by mass spectrometry.

The first such large-scale protein-omic study in humans quantified 42 proteins from blood fractions of individuals from the inCHIANTI study using 20 commercially available protein analysis assays with varying sensitivities and precisions.^12^ Eight *cis* and one *trans* pQTL were identified. More recently, an aptamer-based approach was used to quantify proteins in human plasma, resulting in the identification of *cis*-linked associations for 60 proteins.^18^ 2D gels and mass spectrometry were used to quantify hundreds of the most highly abundant proteins across HapMap cell lines and the Northern Sweden Population Health Study cohort to identify *cis* but not *trans* genetic associations.^14,19,20^ In this report, we developed a standardized protocol using micro-western arrays (MWAs)^21^ and reverse phase protein arrays (RPPAs) to quantify 441 proteins across 68 unrelated Yoruba (YRI) lymphoblastoid cell lines (LCLs) with a panel of antibodies directed at nearly all human transcription factors (TFs) and many disease-related cell-signaling proteins. We then identified pQTLs and compared the genetic architecture underlying mRNA and protein level variation. Our study systematically examined the relationships between pQTLs and eQTLs, replicated the initial discoveries of pQTL associations, and for the first time functionally validated a *trans* pQTL. Our results indicate that novel mechanisms underlying complex disease risk loci are likely to be revealed through further systems-level protein-omic analyses of cells and tissues across human populations.

## Material and Methods

### Cell Lines

Lymphoblastoid cell lines (LCLs, n = 68) derived from unrelated Yoruba individuals in Ibadan, Nigeria, were obtained from Coriell Institute for Medical Research. They have been genotyped at more than 3.1 million SNPs^22^ and had their RNA quantified by expression arrays,^23,24^ exon arrays,^25^ and RNA-seq.^26^ LCLs were cultured in RPMI 1640 (Mediatech)/20 mM l-glutamine (Mediatech) plus 20% FBS (HyClone Laboratories) for the initial passage and then passaged every 48 hr with LCL medium containing 15% FBS. Cell suspensions were transferred to 25 cm^2^ flasks and incubated at 37°C in a humidified 5% CO_2_ atmosphere. Cell lines were maintained at a concentration of 3.5–4.0 × 10^5^ cells/ml and harvested after the fourth passage, if viability was ≥85%.

### Protein Isolation

Three pellets from each of 68 YRI LCLs were independently thawed, cultured, split, pelleted, and stored at −80°C. Pellets from each independent freeze-thaw were resuspended in 1.0 ml of 1.5% SDS lysis buffer (240 mM Tris-acetate, 1.5% w/v SDS, 0.5% w/v glycerol, 5 mM EDTA) containing 50 mM DTT, protease (1 μg/ml aprotinin, 1 μg/ml leupeptin, 1 μg/ml pepstatin), and phosphatase inhibitors (1 mM sodium orthovanadate, 10 mM β-glycerophosphate). To ensure complete protein denaturation, samples were boiled for 10 min, sonicated for 10 min (alternating 30 s on, 30 s off) with a Bioruptor (Diagenode), and concentrated to 5–10 μg/μl in a 96-well microconcentration device with a 10 kDa molecular weight cutoff (Millipore).

### Antibody Screening

The first antibody set comprised 296 previously validated antibodies directed against 200 unique cell signaling proteins.^21,27,28^ The second antibody set represented a completely uncharacterized set of 4,070 antibodies directed against 1,848 unique TFs. Three biological replicates for each of 11–12 individuals were pooled together into 6 pools for screening of these 4,366 rabbit polyclonal antibodies at a 1:1,000 dilution. Printing, gel fabrication, horizontal semidry electrophoresis, transfer, blotting, and scanning were performed as in Ciaccio et al.,^21^ permitting 96 antibodies to be screened over six pooled lysates per MWA. Antibodies were probed in the 800 channel using goat anti-rabbit IR800-conjugated secondary antibodies (1:5,000) (Invitrogen). A validated mouse monoclonal antibody to β-actin (1:1,500) (Cell Signaling 3700) was included on each blot as a printing control and was measured using a goat anti-mouse Alexa Fluor 680-conjugated secondary antibody (1:7,500) (Invitrogen) in the 700 channel. Fluorescence was quantified using LI-COR Odyssey software (v.3.0) by drawing features around the appropriately sized bands for each sample with a fluorescent protein marker (LI-COR 928-40000) acting as a standard for molecular weight. The raw integrated intensities of each feature were background subtracted using the median of the three pixels surrounding the feature as an estimate of local background, the maximum number of pixels permitted by the LI-COR Odyssey image analysis software to be used for local background estimation. These corrected integrated intensities were used to calculate the average background-corrected integrated intensities of replicate spots. Antibodies that displayed a single predominant band of the predicted size of the targeted protein isoform of interest that accounted for >75% of the entire signal of the lane with a signal-to-noise ratio ≥3 were selected for subsequent population-level quantification by RPPAs; antibodies that displayed at least one band of the predicted size of the targeted protein isoform of interest with a signal-to-noise ratio ≥3 but also additional bands were selected for subsequent population-level quantification by MWAs. Antibodies that passed this screen are listed in Table S2 available online.

### RPPA Protein Level Quantification

Four technical replicates of each of three biological replicates of all 68 individuals were spotted using a noncontact piezoelectric microarrayer (GeSiM Nanoplotter 2.1E) onto nitrocellulose membranes (BioRad). Serial dilutions of each of the six pooled lysates used for the original antibody screen and lysates from an A431 cervical carcinoma cell line (which was used as a positive control for antibodies) were also printed for each array to ensure linearity of antibody signal. Features with a background-subtracted integrated intensity <0 or signal-to-noise ratio <3 (*z* test p > 0.05) were identified in each array and excluded from further analysis. The distributions of background-corrected integrated intensities for all features on each array were first log_2_-quantile normalized using the limma^29^ package in R to correct for overall antibody hybridization efficiency differences in the signal. The relative level of a given protein for a sample was then quantified using the linear model *y*_*jp*_ *∼μ*_*jp*_ + λ_*j*_ + *e* (Equation 1), where *μ*_*jp*_ is the log-quantile-normalized, background-corrected integrated intensity of sample *j* on array *p*, and λ_*j*_ is the effect due to sample *j* across all arrays in a print (due to differing amounts of total protein spotted on the array for each sample), estimated by median_*j*_ (*μ*_*jp*_). Notably, we performed an extension of median loading normalization^30^ and did not normalize to housekeeping proteins such as β-actin or α-tubulin as is typically performed with traditional western blotting to correct for sample load, because interindividual β-actin mRNA levels varied by two orders of magnitude in the RNA-seq data. Odyssey output text files were parsed in Python and quantified and normalized in R.

### MWA Protein Level Quantification

Three technical replicates of each of the three biological replicates of all 68 individuals were spotted as above onto polyacrylamide gels. Gel fabrication, horizontal semidry electrophoresis, transfer, and scanning were performed as in Ciaccio et al.^21^ with the exception of separating each blot into four quadrants rather than using a 96-well gasket. This method allowed for 68 samples to be quantified with a single antibody in triplicate from each of four quadrants. Feature extraction and data normalization were performed the same as with RPPAs. For antibodies that produced multiple significant bands (signal-to-noise ratio > 3), all isoforms were quantified and their relative molecular weights recorded. The level of a given protein for an individual was quantified using the above linear model (Equation 1) with the addition of a batch term (β) to correct for global intensity distribution differences across multiple MWAs for the same antibody. We averaged measurements across replicates *within* platforms for the same antibody across the entire population. For replicates *across* platforms, we selected the platform that yielded the highest median background-corrected integrated intensity. To reduce the inflated effect of technical noise because of low antibody signals and provide more accurate interindividual protein level measurements, antibodies in the bottom deciles of median background-corrected integrated intensities or in the top deciles of technical coefficients of variation for either platform were flagged and eliminated from further analyses.

### Quality Metrics of the Protein Measurements

To correct for differences in the total amount of protein deposited for each sample for each array, we estimated a sample load effect by regressing out the median protein measurement for each sample. This measurement was highly correlated with the first principal component of the protein data, as the overall concentration of each sample was directly related to the amount of each protein (*R*^*2*^ > 0.95). To assess the quality of our protein data, we plotted the coefficients of variation for each antibody quantified versus the median signal-to-noise ratios and background-corrected integrated intensities (Figure S1). Similar to the effect observed with expression microarrays, we observed relatively high technical variation for antibodies of low fluorescence signal and a trend toward decreased variation as fluorescence signal began to exceed the noise floor of our proteomic platform. Therefore, we removed protein measurements in the bottom quartiles of signal-to-noise ratio and background-corrected integrated intensity and the top quartile of coefficient of variation. The application of these filters reduced the effect of technical variation on our later inferences. We observed a median σ of 0.47 between interindividual protein levels quantified by RPPAs and MWAs from seven antibodies quantified across both platforms (example shown in Figure S2). Comparatively, the median σ between expression arrays and RNA-seq for any given transcript across the same population of YRI individuals has been shown to be approximately 0.12.^26^

To validate that the antibodies generated against epitopes within each protein were targeting the protein of interest, we performed two analyses. First, for the 57 pairs of antibodies directed at different epitopes for the same protein that passed our screen, we tested for correlated measurements between interindividual protein levels measured by both antibodies. We observed that 44 of the 57 had correlated measurements (ρ > 0). Discordance between multiple antibodies to the same protein could be because of technical variation or differential isoform levels, because each epitope is directed to a unique region of each protein. Second, approximately 50 amino acids surrounding known antibody epitopes (because exact epitope information was proprietary) were remapped to the human genome (UCSC Genome Browser build hg18) using BLAT^31^ and epitopes that contained at least one nonsynonymous SNP from dbSNP (release 132)^32^ or matched multiple regions in the genome with at least 95% identity were flagged but retained, because the proprietary nature of epitope disclosure prevented us from knowing which ∼5–8 amino acid fragment of the 50 amino acids was used to create the antibody.

### Gene Expression Data

Expression array data for 53 individuals included in our study from Illumina’s human whole-genome expression arrays (WG-6 v.1) from Stranger et al.^24^ were downloaded from Gene Expression Omnibus (GSE6536). Probes were remapped to the human genome (UCSC Genome Browser build hg18) using BLAT^31^ and probes that mapped to a single location with less than 100% sequence identity or mapped to multiple locations with up to two mismatches were discarded. We then excluded probes that contained at least one SNP in dbSNP (release 132)^32^ or our imputed common SNP genotypes for our cohort or overlapped copy-number variants in the YRI population.^33^

Exon array data for 52 individuals overlapping our study from the Affymetrix GeneChip Human Exon 1.0 ST Array platform from Zhang et al.^34^ were downloaded from Gene Expression Omnibus (GSE9703). Probes were mapped to UCSC Genome Browser build hg18 and probes containing at least one SNP were removed from probe set signal intensity files. Gene-level expression of transcript clusters was summarized with RMA^35^ and averaged within unique Ensembl gene annotations.

RNA-sequencing data were obtained for all individuals in our study from Pickrell et al.^26^ Gene expression values were calculated as the number of GC-corrected reads mapping to a gene in an individual, divided by the length of the gene in kilobases and the number of mapped reads across all lanes for that individual in millions (RPKM).

### Cellular Covariates and Hidden Confounders

We quantified the EBV copy number in all LCLs. EBV copy number was assessed with a Taqman Gene Expression Assay (Pa03453399_s1). Intrinsic growth rates for each cell line from Im et al.^36^ and baseline ATP and mitochondrial DNA levels from Choy et al.^37^ were also included in the analyses. To identify potential additional unobserved confounders, we applied surrogate variable analysis (SVA) to the matrix of 68 × 3 protein level measurements after including the effects of known nongenetic confounders to identify 16 additional significant surrogate variables.

### Covariate Modeling

For each protein measurement, we constructed a linear mixed effects model *y* ∼*p* + *E + M + A + G + S + P* + *T|I* + *SV*_*i..n*_ + *e*, in which *p* is the array- and sample-load-normalized integrated intensity for all biological replicates in the population, *E* is the fixed effect of individual EBV copy number, *M* is the fixed effect of individual mitochondrial DNA copy number, *A* is the fixed effect of individual baseline ATP levels, *G* is the fixed effect of individual intrinsic growth rate, *S* is the fixed effect of individual sex, *P* is the fixed effect of individual phase, *T*|*I* is the random thaw effect per individual, *SV*_*i..n*_ are the effects of a matrix of 16 significant surrogate variables, and *e* is the residual error. The model was fitted to each protein by residual maximum likelihood using the lmer function in the R package lme4 (v 0.999999-0). Fixed effect p values for covariates were estimated using the pamer.fnc function in the LMERConvenienceFunctions package (v.1.6.8.3). The significances of covariate effects were assessed by estimating false discovery rates using Storey’s q value method.

### Genotype Data

HapMap genotypes were obtained from the 1000 Genomes June 2011 phase I low-pass whole-genome SNP genotype release and transformed to UCSC Genome Browser (hg18) coordinates. Missing values were imputed by BIMBAM (v.1.0) using the default parameters to derive mean imputed genotypes. SNVs with MAF < 0.05 and SNVs with significant deviation from Hardy-Weinberg equilibrium (Fischer’s exact test, p < 0.001) were excluded, reducing the set to 9,345,571 SNPs and indels for association analyses.

### Association Analyses

For each protein and transcript trait, interindividual levels were inverse normal transformed and tested for association with all markers genome-wide. Association testing was performed by linear regression implemented in Python and R using custom scripts. For each trait, we selected the most significantly associated SNV within each recombination window, defined by splitting the genome into 25,307 blocks flanked by >10 cM/Mb recombination rates estimated from HapMap.^22^ All SNV-protein associations with p < 10^−4^ for proteins with more than one biological replicate were validated with the linear mixed-effects model *y* ∼*p* + *G* + *T|I* + *e* with a fixed genotype variable, *G*.

### Significant Associations

We performed genome-wide permutations to assess the significance of the association results. We permuted the 468 protein values for each biological replicate for all individuals, performed genome-wide association on the permuted and normalized phenotypes, and repeated this procedure for three replicates, selecting each time the best signal per phenotype. Permuted SNV-protein associations with p < 10^−4^ were tested with a linear mixed-effects model as above. False discovery rate (FDR) was calculated as the fraction of significant hits in the permuted versus the observed data at a given p value threshold. FDR was calculated separately for *cis* and *trans* pQTLs. Results are presented at FDRs of 5% and 20%, meaning that an estimated 5% and 20% of the pQTLs correspond to false positives, respectively. We chose to perform association analyses on protein and mRNA measurements without covariate and SV correction because correction for known covariates, SVs, or both did not improve RNA-protein correlations (p > 0.05, Wilcoxon rank sum test). We observed fewer *cis* and *trans* pQTLs at an FDR < 0.20 after correction (suggesting that we might be discarding some fraction of genetic variation associated with protein level variation, as has been previously demonstrated in methods to optimize *cis* eQTL discovery by iteratively removing PCs to maximize the number of eQTLs discovered^26,38^) and to be consistent with all previous pQTL studies to date^10–12,14,19,20^ to allow more direct comparison of our results. The association analyses and FDR calculations were performed for all autosomal surrogate variables (n = 16), protein values, and genes in the mRNA expression data sets.

### Enrichment of Specific Types of SNVs in pQTLs and eQTLs

The annotation of all SNVs was performed using SeattleSNP Annotation 129. For each unique annotation (“coding-synonymous,” “intergenic,” “intron,” “missense,” “near-gene-3,” “near-gene-5,” “nonsense,” “splice-3,” “splice-5,” “utr-3,” and “utr-5”), we used a Fisher exact test to test the null hypothesis that the fraction of that annotation type in either recombination-block-filtered eQTLs or pQTLs for overlapping gene models at p < 10^−4^ was equal to the fraction in all annotated SNVs.

### Genome-wide Association Study Results and Enrichment Analyses

All SNPs published by 02/01/2012 were downloaded from the catalog of GWASs maintained by the NHGRI and filtered for 5,570 common variants (MAF > 5%) in the YRI samples examined. For overlap with eQTLs and pQTLs, we considered all SNPs in linkage disequilibrium (LD) (R^2^ > 80%) with the complex-trait-associated SNPs and filtered for common variants (MAF > 5%) in the YRI samples examined. To determine the enrichment for SNPs associated with each complex trait to be eQTLs or pQTLs, we focused on only the 7,222 primary-trait-associated SNPs before LD imputation to correct for LD-driven inflation of enrichment results. We then generated 1,000 randomized SNP sets each of size 7,222 and matched on MAF distribution by proportions in discrete 5% MAF bins. For each set, we determined the number of eQTLs and pQTLs at p < 10^−4^ for traits with at least three observed expression QTL overlaps and derived an empirical p value by comparing the proportion of random simulations in which the number of random overlaps exceeded the observed overlap.

### siRNA Knockdown

LCLs were seeded at a density of 550,000 cells/ml 24 hr before nucleofection. Amaxa’s Cell Line 96-well Nucleofector Kit SF (Lonza) was used to perform the transfection. Cells were centrifuged at 90 × *g* for 10 min at room temperature and resuspended at a concentration of 1,000,000 cells in 20 μl of SF/supplement solution (included in SF Kit Lonza Catalog #V4SC2096) and 2 μM final concentration of AllStars negative Control siRNA labeled with Alexa Fluor 488 (QIAGEN) or a pool of siRNA (QIAGEN) (Table S1). The cells were nucleofected using Amaxa’s DN-100 program. Cells were allowed to rest for 10 min before the addition of prewarmed (in 37°C water bath for a minimum of 20 min) RPMI media and then another 5 min after the addition of warm RPMI media. Cells were then plated for protein harvest. Cells were harvested at 24 and 48 hr postnucleofection for protein measurement by MWAs. Protein levels were quantitated as above with three technical replicates per individual per time point and normalized within an individual across time points to the relative β-actin protein levels. Percentage knockdown was then calculated by dividing the relative targeted protein levels in the targeted siRNA sample by those in the scrambled siRNA control sample for each time point. A knockdown was declared significant if protein levels were reduced after knockdown by greater than two times the percentage standard error (p < 0.05).

## Results

### Study Design and Protein Quantification

**Figure 1:**
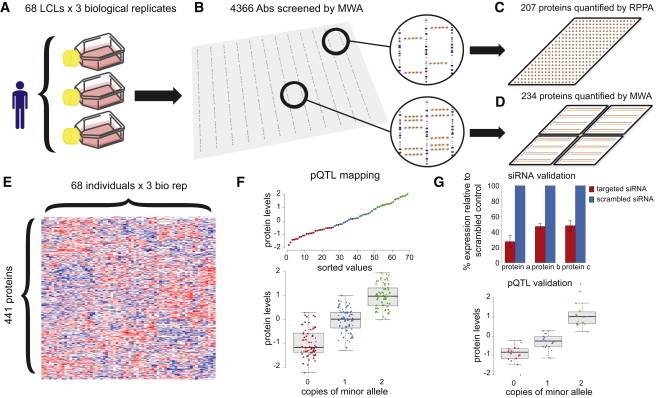
Schematic Representation of the Experimental Design (A) Three biological replicates from each of 68 YRI lymphoblastoid cell lines were passaged, pelleted, and lysed. (B) These lysates were aggregated into six pools for screening 4,366 polyclonal antibodies at a 1:1,000 dilution. (C and D) Antibodies that produced only a single (C) or at least one (D) predominant band the size of the target protein with a signal-to-noise ratio ≥3 were subsequently scaled up for population-level quantification by RPPAs and MWAs, respectively. (E) Sample load and batch effects were then regressed out to derive a final matrix of 68 individuals by 441 protein levels. (F) Residuals were inverse normal transformed and associated with 9,345,571 SNVs genome-wide to identify pQTLs. Triangles, circles, and diamonds correspond to biological replicates. (G) A random subset of antibodies was validated by siRNA knockdown and pQTLs were tested for replication in an independent cohort of 17 unrelated YRI LCLs.

To characterize the genetic architecture of a targeted subset of proteins in humans, we measured protein levels from three independently cultured replicates (hereafter termed biological replicates) of 68 HapMap YRI LCLs for which genotypes, mRNA expression,^24,26,34^ and pharmacologic data^39^ were available. A common problem encountered with contemporary affinity-based protein-omic studies^17^ is the lack of well-validated antibodies at economical prices. We therefore took a two-pronged approach to maximize our ability to collect high-quality protein data and to comprehensively collect data on poorly characterized and lowly expressed transcription factors (TFs). Our antibody set comprised 4,366 antibodies directed against 1,848 unique TFs and 200 unique cell signaling proteins. We screened these antibodies against six pools of lysates comprising 68 YRI individuals (Figures 1 and S3; Table S2). A total of 207 antibodies produced a single predominant signal at the predicted molecular weight and were subsequently used to quantify protein levels via the RPPA approach (representative array shown in Figure S4 and Table S3). Because RPPAs lack the ability of MWAs to electrophoretically separate proteins, sample throughput and image analysis are more rapid. However, much higher selectivity antibodies are required for RPPAs than for MWAs to obtain meaningful data.^40^ A total of 234 antibodies that produced signals in addition to those at the predicted molecular weight were measured at the population level via MWAs (Figure S5; Table S4). This approach ultimately allowed us to quantify protein levels from 441 antibodies (341 TF and 100 signaling) directed at 391 unique protein isoforms (Table S5) across 68 LCLs cultured on three independent occasions.

We established the quantitative accuracy of our approach by several independent methods. First, to address sources of technical variation in our measurements, we established methods to normalize and filter the protein data as described in the Methods. Second, we observed that 13 pairs of antibodies targeting different epitopes for the same proteins resulted in significantly correlated measurements for their intended targets across all individuals (p < 0.05) (Figure S6). Third, we observed a strong preservation of interindividual rank order for the same protein quantified by both RPPAs and MWAs (example illustrated in Figure S2). Fourth, we verified that the antibodies faithfully reported on the levels of their intended targets by performing siRNA knockdown of 18 proteins, 15 of which were randomly selected and 3 of which were pQTL targets. Of the 18, 17 exhibited a significant reduction in protein levels relative to a scrambled control in at least one YRI LCL at one time point (Table S6).

### Low Preservation of Rank Order between mRNA and Protein Levels across All Individuals

**Figure 2:**
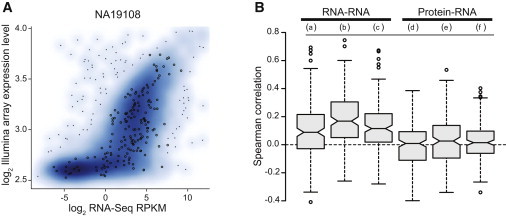
Relationship between Transcript and Protein Levels (A) Correlation between Illumina array and RNA-seq expression measurements within individuals. Log_2_-transformed expression estimates derived from the array are plotted with a smoothed density against log_2_-transformed RPKMs derived from RNA-seq. Explicit circles denote genes with corresponding protein-level quantifications. (B) Boxplots of Spearman correlation coefficients between RNA-sequencing, Affymetrix exon array probe sets, Illumina expression array probe sets, and antibodies targeted to the same gene for all 68 individuals. The boxplots are labeled as follows: (a) RNA-seq versus exon arrays, (b) exon arrays versus expression arrays, (c) RNA-seq versus expression arrays, (d) protein versus expression arrays, (e) protein versus exon arrays, and (f) protein versus RNA-seq.

We first compared the correlations between mRNA expression levels within individuals measured by three expression platforms: Affymetrix exon arrays,^25^ Illumina expression arrays,^24^ and RNA-sequencing (RNA-seq).^26^ We observed a strong correlation between these independent measures of transcript abundance for all genes (Figure 2A; RNA-seq versus Illumina expression array, median ρ = 0.67; RNA-seq versus Affymetrix exon array, median ρ = 0.82; Illumina expression array versus Affymetrix exon array, median ρ = 0.62). These observations support previous reports that have demonstrated similarly highly correlated mRNA expression measurements between RNA-seq and expression array technologies when measuring mRNA expression from a single individual (ρ = 0.60–0.77).

However, for expression QTL analysis, the more relevant comparison is how well expression levels correlate for each gene measured across individuals. We examined the preservation of rank order between 173 overlapping gene-level protein and mRNA measurements across the 52 individuals that were examined in each study (Figure 2). Notably, the correlation of interindividual mRNA expression measurements was low across mRNA expression platforms and laboratories (median ρ = 0.09–0.17), consistent with results from a previous eQTL analysis on this cohort using all genes (median ρ = 0.12).^26^ The correlation between mRNA levels was significantly higher between microarray platforms (median ρ = 0.17) than between microarrays and RNA-seq (median ρ = 0.10, p = 2.80 × 10^−4^, Wilcoxon rank sum test), indicating that either biological or platform variance contributed substantial variability to previous mRNA expression studies. Similarly to previous observations in yeast and mice,^10,11,15^ little correlation was observed between transcript and protein levels within genes, across individuals (exon array median ρ = 0.03, expression array median ρ = 0.01, RNA-seq median ρ = 0.02) (Figure 2B). Although global mRNA and protein levels were not strongly correlated across individuals, they were enriched to be correlated with 12% of genes displaying significant preservation of interindividual rank order between mRNA and protein levels, even in the presence of biological variation associated with the propagation of cells across different laboratories.

Several cellular characteristics including intrinsic growth rate, ATP levels, and EBV copy number have previously been shown to associate with gene expression levels and cellular phenotypes measured in LCLs.^37,41,42^ mRNA expression data sets have previously been adjusted for these cellular covariates to increase the ability to observe relationships between genotypes and mRNA expression levels.^8,26,37^ We surmised that these cellular variables might also be related to protein levels. We therefore tested for association between interindividual variation in these variables and with protein levels. We identified 197 protein variants that were nominally associated with at least one of six variables (Table S7). At an FDR of 5%, we found that 36 proteins were associated with intrinsic growth rate,^35^ 28 proteins were associated with baseline ATP levels, and 21 proteins were associated with EBV copy number. Levels of phospho-S6 ribosomal protein (RPS6) and structural maintenance of chromosomes protein (SMC1A) were negatively correlated with cell growth (*R* = −0.33, p = 1.86 × 10^−6^ and *R* = −0.19, p = 2.62 × 10^−4^, respectively). Notably, we found that β-actin and α-tubulin protein levels were positively correlated with intrinsic growth rate (*R* = 0.12, p = 1.36 × 10^−5^ and *R* = 0.02, p = 0.04, respectively), suggesting that their use in total protein load normalization as housekeeping proteins, rather than the median sample load normalization we performed, would have resulted in an erroneous adjustment for intrinsic growth rate differences between cell lines.

Nominally associating with 21% of protein-level and 25% of RNA-seq-derived mRNA-level measurements, intrinsic growth rate was correlated with the highest number of mRNA and protein levels (Table S8). Indeed, of the 18 significant surrogate variables (SV) identified in the protein data set, the first SV was significantly associated with intrinsic growth rate (p = 0.03) and EBV copy number (p = 0.05), and the third SV was associated with intrinsic growth rate (p = 7.0 × 10^−4^), underscoring the high degree of association between global protein levels, intrinsic growth rate, and EBV copy number in LCLs.

### pQTL Mapping and Replication in an Independent Cohort of LCLs

**Figure 3:**
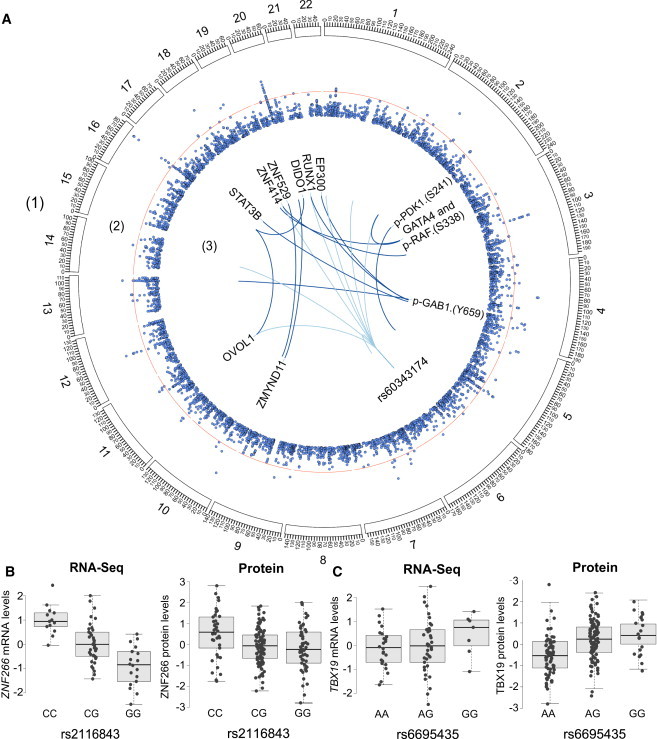
Genetic Variants Affecting Protein Levels Globally and in *cis* (A) Circos plot for the pQTL association results. The rims, in order from outside inward, are (1) a karyotype defining chromosome coordinates, (2) a Manhattan plot of the –log_10_(*P*) for each pQTL identified at p < 10^−4^ (for plotting clarity) with the red line designating p = 10^−8^, and (3) the top 12 (constrained due to space) significant pQTLs (p < 10^−10^) and the top master regulatory pQTL SNV rs60343174. The innermost network depicts spokes between pQTLs and their regulated genes, with dark blue spokes depicting the top pQTL interactions (such as rs6834 with DIDO1 protein levels) and light blue spokes depicting proteins associated with the top master regulatory pQTL. (B) RNA-seq and protein measurements (y axis) for each sample plotted by SNP genotype for a replicated *cis* eQTL for ZNF266. Error bars represent 95% confidence intervals. (C) RNA-seq and protein measurements for TBX19 versus SNP genotype for a representative *cis* pQTL.

We performed global pQTL mapping by testing for association between the 441 protein level measurements and genotypes at 9,345,571 single-nucleotide variants (SNVs). At an FDR < 0.20, we identified 12 *cis* pQTLs (here defined as within 1 Mb upstream of the transcription start site [TSS] to 1 Mb downstream of the transcription end site [TES] of the RefSeq gene model) and 160 *trans* pQTLs (corresponding to p < 3.26 × 10^−6^ for *cis* and p < 3.42 × 10^−8^ for *trans*) (Table S9). The most significant *cis* and *trans* pQTLs at an FDR < 0.05 corresponded to 18 unique RefSeq gene models (Table 1). This observation of larger numbers of *trans* than *cis* pQTLs corroborates similar observations from previous pQTL studies in yeast^11^ and mice.^15^ We constructed Circos plots to visually depict significant associations between SNVs, protein levels (Figure 3), and mRNA expression levels (Figure S7). The 12 most significant pQTLs as well as the SNP associated with the most protein levels are illustrated in Figure 3; similarly, the ten most significant eQTLs as well as the SNP associated with the most mRNA transcript levels are shown in Figure S7. For example, rs60343174 was significantly associated with the relative levels of eight proteins, including OVOL1, ZNF414, and RUNX1 (Figure 3A).

To further validate the pQTL associations, we randomly selected 20 proteins for quantification in three biological replicates of a separate, unrelated cohort of 17 YRI LCLs from Coriell and tested for replication of pQTLs for all associations discovered at p < 10^−4^ (Table S10). Of the eight pQTLs (FDR of 0.20) that were associated with proteins quantified in our replication cohort, seven had consistent effect directions, and for two, the replications were also nominally significant (rs145614393 and ZMYND11; rs60664312 and DIDO1). The rs60664312-DIDO1 association was the largest effect size pQTL in the discovery cohort, whereas the rs145614393-ZMYND11 association was of moderate effect size (Table S10). None of the 30 pQTL SNVs identified at an FDR < 0.05 was also significantly associated with cellular covariates, suggesting that cellular covariates did not confound pQTL identification. Additionally, we observed more pQTLs before regressing out known covariates and/or unknown confounding effects as estimated by surrogate variable analysis. We therefore chose to perform all further analyses using the unadjusted protein values, identical in approach to all previous pQTL studies to date.^10–12,14,19,20^ Although no RNA-seq eQTLs identified at an FDR < 0.05 correlated at similarly stringent p values in our protein data as pQTLs, the most significant *cis* eQTL identified at an FDR < 0.05 was also a nominally significant pQTL: an association between variation at rs2116843, an intergenic SNP approximately 20 kb upstream of the transcription start site (TSS) of ZNF266 (MIM 604751), and variation in ZNF266 expression at the mRNA (p = 1.54 × 10^−9^, β = −0.65) and protein level (p = 0.003, β = −0.21) (Figure 3B). This observation provided an example of a genetic variant that associated with both the mRNA and protein level, as would be expected if the genetic variant causally influenced mRNA and subsequently influenced protein level variation. We also observed another, more significant *cis* pQTL for ZNF266, rs7256500 (p = 1.68 × 10^−7^, β = 0.37), which is an intronic SNP located approximately 0.8 Mb upstream of the TSS of ZNF266 within MYO1F (MIM 601480) that was not in LD with rs2116843 (*r*^*2*^ = 0.04). This pQTL was not significantly associated with mRNA expression levels, although the effect trend was in the same direction (p = 0.15, β = 0.18). Additionally, rs6695435, an intergenic variant located 4 kb upstream of the TSS of TBX19 (MIM 604614), had a subtle but not significant association with mRNA expression as measured by RNA-seq (p = 0.28, β = 0.14) (Figure 3C), but a significant association with concordant effect direction on TBX19 protein levels (p = 5.17 × 10^−6^, β = 0.33) and was one of the closest *cis* pQTLs to the TSS of the gene model that we identified.

We observed significantly more *trans* associations with protein levels than with mRNA levels (160 *trans* pQTLs for 78 unique proteins versus 0 *trans* eQTLs at an FDR = 0.20). The most significant pQTL identified was the association between SNP rs60664312 and DIDO1 protein levels (p = 5.55 × 10^−17^, β = 0.65). We observed an intergenic indel on chromosome 6 (rs60343174) that was associated with eight different protein levels at p < 10^−4^ and that was most significantly associated with LMX1A (p = 5.66 × 10^−9^, β = 0.51) (Figure 3A). Consistent with results from Wu et al.,^14^ we observed that measurements of protein variation were not significantly influenced by posttranslational modifications for the proteins for which both pan- and phospho-specific validated antibodies were available. However, three of the most significant *trans* pQTLs were intergenic variants affecting the levels of a phosphorylated isoform of three proteins but not their overall abundances as inferred using pan-specific antibodies (p > 0.05): rs7331659 and p-Gab1 (Y659), rs751473 and p-Raf (S338), and rs2016050 and p-PDK1 (S241) (Figure 3A). This observation represents, to our knowledge, the first evidence of genetic variants associated with the phosphorylated version of a protein and represents a first step toward identifying common genetic variants associated not only with protein levels, but also with their modification states, which often serve as proxies for their activation states.

### Comparison of Genetic Variants Associated with mRNA and Protein Levels

**Figure 4:**
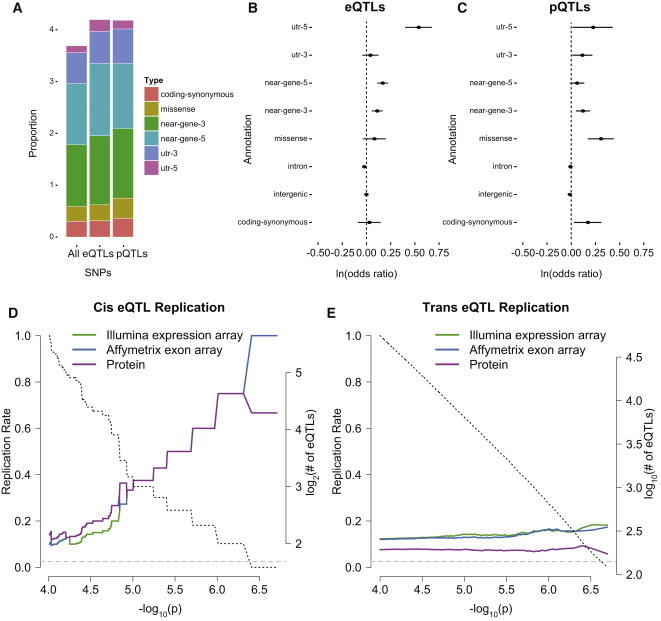
Comparison of the Genetic Regulation of Transcript and Protein Levels (A) Proportion of annotations other than “intron” and “intergenic” for all SNVs, eQTL SNVs, and pQTL SNVs. (B and C) Log odds that an eQTL (B) or a pQTL (C) is a particular annotation type versus all SNVs in the study. (D and E) *cis*-eQTL (D) and *trans*-eQTL (E) replication rate (y axis) is depicted as a function of the p value in transcriptome sequencing (x axis) for Illumina expression array (green), Affymetrix exon array (blue), and protein data (purple). The black dashed lines denote the number of eQTLs at each discovery p value. The gray dashed line denotes the null expectation of replication rate at p < 0.05 with concordant effect direction.

We next examined the functional classifications, locations, and reproducibility of genetic loci affecting protein abundances. We compared the newly identified pQTL loci with eQTL loci that we identified using RNA-seq expression for the 373 genes with overlapping protein and mRNA measurements (Figure 4). We first compared the relative proportions of annotations of eQTLs and pQTLs versus all SNVs used in our study (Figure 4A; Table S11). eQTLs were significantly enriched near the 5′ and 3′ ends of genes and at the 5′ UTR (p = 6.63 × 10^−9^, p = 1.49 × 10^−3^, and p = 6.27 × 10^−13^, respectively) and depleted in introns (p = 1.42 × 10^−3^) (Figure 4B). This finding is consistent with observations from previous global eQTL studies that eQTLs tend to cluster near the TSSs of genes^24,26^ and in exons relative to introns.^43^ pQTLs were enriched near gene 3′ ends (1.14 × 10^−3^), in 5′ or 3′ UTRs (p = 0.03, p = 0.03), in synonymous coding variants (p = 0.02), and notably, in missense variants (p = 1.58 × 10^−5^) (Figure 4C). These observations indicated that genetic variants associated with protein level variation might involve protein stability or miRNA-mediated regulation of mRNA translational efficiency. We next examined the reproducibility of eQTLs across platforms and at the protein level (defined as a nominal p < 0.05 with a concordant effect direction) as a function of the p value of the discovery association in RNA-seq. We observed that *cis* and *trans* eQTLs discovered at p < 10^−4^ were more likely to replicate across platforms and as pQTLs than expected by chance (Figures 4D and 4E). All four of the most significant *cis* eQTLs (p < 10^−6^) replicated by both the Illumina expression array and Affymetrix exon array, and three were nominally significant *cis* pQTLs. Two of these *cis* eQTLs were located in adjacent recombination blocks and associated with ZNF266; indeed, despite being split by an average 29 cM/Mb recombination rate across the 20 kb gene transcription region centered at chr19: 9,348,911,^22^ these SNPs remained in high LD (*r*^2^ = 0.99). However, *trans* eQTLs failed to replicate well across mRNA expression platforms or between mRNA and protein measurement platforms (Figure 4E). This observation was consistent with previously reported examples demonstrating the difficulty in replicating *trans* eQTLs.^8,44^ Only 1 of the 10 *cis* pQTLs (rs7256500 and ZNF266) and 6 of the 49 *trans* pQTLs identified at an FDR < 0.20 replicated at the mRNA level, indicating that few genetic variants that were strongly associated with protein levels were also associated with mRNA expression (Figure S8). These observations suggested that many of these genetic variants might affect protein levels independently of their effect on transcript levels.

### The Lysyl-tRNA Synthetase KARS Underlies a *trans* pQTL for DIDO1

**Figure 5:**
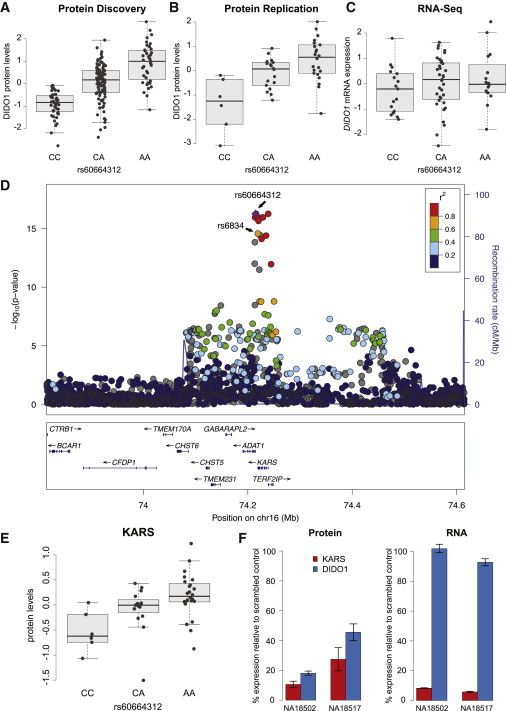
Identification and Validation of a *trans* pQTL for DIDO1 (A–C) Protein discovery (A), replication cohort (B), and transcriptome sequencing expression (C) measurements (y axis) for each sample plotted by SNP genotype for a replicated *trans* pQTL for DIDO1. (D) Regional association plot of the genic region associated with DIDO1. The –log_10_(p value) for SNVs in this region (left y axis) and recombination rate (right y axis) are depicted with respect to genomic position (x axis) using LocusZoom.^45^ SNVs in LD with the most associated SNP are plotted according to the color scale. (E) KARS protein levels versus genotype in the replication cohort of 17 YRI LCLs. (F) DIDO1 protein, but not mRNA, expression levels were significantly reduced 24 hr after siRNA knockdown of KARS. Error bars indicate the SEM of three technical replicates for each condition.

Above, we noted the identification of the SNP rs60664312 as a significant *trans* pQTL for Death Inducer-Obliterator 1 protein (DIDO1) (p = 5.55 × 10^−17^, Figure 5A) and subsequently validated the *trans* pQTL relationship for DIDO1 in a replication cohort (Figure 5B). This pQTL was not an eQTL (p > 0.05, Figure 5C) based on previous mRNA expression data. DIDO1 is involved in limb development and the induction of apoptosis in mice.^46^ This pQTL exists in a linkage disequilibrium (LD) block that contains the genes *ADAT1* (MIM 604230), *KARS* (MIM 601421), and *TERF2IP* (MIM 605061) (Figure 5D) but was not described as a *cis* eQTL for any of these genes in previous mRNA expression studies (p > 0.05). However, rs60664312 is located 4 kb upstream of the TSS of KARS and is in high LD (*r*^*2*^ = 0.87) with rs6834 (RefSeq accession number NP_001123561.1; p.Thr623Ser, DIDO1 pQTL p = 2.66 × 10^−15^, β = −0.63), a nonsynonymous variant for KARS that would be predicted to influence the ability of the protein to be phosphorylated by a protein kinase. To examine whether this variant was expected to be functionally significant, we assessed Genomic Evolutionary Rate Profiling (GERP),^47^ and Sorting Intolerant From Tolerant (SIFT)^48^ scores. GERP scores address nucleic acid sequence preservation during mammalian evolution. Scores >2 are considered “constrained” and are more indicative of a deleterious polymorphism. SIFT scores attempt to predict whether an amino acid substitution will affect protein function based on the degree of conservation of amino acid residues in sequence alignments derived from closely related sequences, with scores <0.05 being considered “deleterious.” RefSeq NP_001123561.1 (p.Thr623Ser) was predicted to be extremely deleterious at the nucleic acid level (GERP score = 5.82), despite being predicted to be well tolerated at the protein function level (SIFT score = 0.47). We next quantified KARS protein levels in a replication cohort and examined whether the SNP rs60664312 was a pQTL for KARS. We observed a significant correlation with KARS protein levels for both rs60664312 and rs6834 (p < 0.002 for both comparisons) in the same effect direction as DIDO1 levels. The minor allele of rs6834 was associated with higher DIDO1 and KARS protein abundances, respectively (Figure 5E). To examine the causality of this relationship between DIDO1 and KARS protein levels, we performed siRNA-mediated knockdown of KARS and observed a concomitant reduction in DIDO1 protein levels (p < 0.001) without effects on DIDO1 mRNA levels (p > 0.05) as measured by qRT-PCR (Figure 5F). To address whether knockdown of KARS would affect DIDO1 protein levels specifically, we included ZNF569 as a negative control and observed no reduction in ZNF569 protein levels after KARS knockdown (Table S6). In summary, we identified a SNP that appeared to influence DIDO1 protein levels through the abundance and activity of KARS in a manner that was independent of underlying KARS mRNA levels and that was detectable only through measurement of protein levels.

### Overlap of Complex Trait QTLs and pQTLs

Previous studies have demonstrated that common genetic variants associated with complex traits significantly overlapped with eQTLs.^7^ Genetic risk factors have often been assumed to influence complex traits through their effects on mRNA expression. However, many posttranscriptional mechanisms exist that could influence phenotypic variability through unique effects on protein abundances. To explore this notion, we tested for overlap between pQTLs identified here and complex-trait-associated SNPs in the NHGRI GWAS catalog. Of the 7,222 SNPs associated with 612 complex phenotypes and diseases at p < 10^−8^, 197 overlapped with at least one pQTL at p < 10^−4^ (Table S12). We identified several notable overlaps between pQTLs and complex trait SNPs offering potential insights into the molecular mechanisms underlying these phenotypes (Table 2). For example, we identified an intergenic pQTL associated with HOXB7 levels (rs991258) and two intergenic pQTLs (rs731905 and rs9398038) for HOXB10 levels that overlapped with SNPs that were previously found to be associated with hip geometry,^49^ height,^50^ and primary tooth development.^51^ HOXB7 and HOXB10 are members of the homeobox gene family and function to regionalize the embryo along its major body axes.^52^

## Discussion

We utilized the MWA approach to screen more than 4,300 antibodies. From this screening effort we identified 441 antibodies against 391 unique proteins that we used for subsequent quantification of relative protein levels within a population of YRI LCLs. We performed comparative analysis of the relationship between genetic variation and between subsets of the transcriptome and proteome. We replicated and functionally validated a significant *trans* pQTL from our proteomic association analyses. Because of the difficulties in comparison of existing large-scale genomic and proteomic data sets, our study represents one of the first to examine large-scale relationships between genome variation, mRNA expression, protein levels, and protein modifications in human cells.

Our data complement studies that have been previously undertaken in yeast and mice^11,15^ that indicated relatively low interindividual correlations between mRNA and protein levels. Compared to studies conducted in yeast or mice, we observed a slightly lower protein-transcript concordance, but still approximately zero (median ρ = 0.02 for humans versus 0.07 for yeast). This lack of correlation between mRNA and protein levels could partially be because of technical variation, particularly limitations in accurately quantifying lowly expressed mRNA transcripts or protein abundances. However, the comparative analysis of three separate mRNA expression platforms with a MWA-derived protein data set enabled us to better address this issue. The general lack of correlation between mRNA and protein levels could be explained biologically by molecular mechanisms such as mRNA translation efficiency, protein folding, protein stability, protein assembly into complexes, transport and localization, or covalent modification, all of which affect proteins independently from mRNA transcripts. Taken together, our data suggest that cells may have the capacity to buffer effects on protein that genetic variation has on mRNA expression levels.

We identified 12 *cis* and 160 *trans* pQTLs at an FDR < 0.20 (compared with 11 *cis* and 0 *trans* eQTLs at the same threshold). Although up to two thirds of *cis* eQTLs also were also nominally significant *cis* pQTLs, the majority of *cis* pQTLs were not nominally significant *cis* eQTLs. *trans* eQTLs and pQTLs did not replicate well across any platform. This result is consistent with previous studies that have demonstrated difficulty in reproducing *trans* eQTLs across mRNA expression platforms.^8,53^ However, 16 of 18 of the most significant *trans* pQTLs (p < 10^−7^, FDR = 0.29) had reproducible effect directions in the additional cohort of unrelated YRI LCLs that we examined during the functional validation phase of our project. We identified similar numbers of *cis* eQTLs versus *cis* pQTLs for the same gene models examined by both platforms (11 *cis* eQTLs for 10 genes versus 12 *cis* pQTLs for 11 genes, FDR = 0.20), consistent with previously observations of ∼4% of genes having *cis* eQTLs in the same cohort.^26^ Consistent with Foss et al.^11^ in yeast, we identified a larger number of *trans* pQTLs than *trans* eQTLs. These results suggest that genetic variants affecting mRNA levels tend to have stronger effects in *cis*, whereas variants affecting protein levels tend to have stronger effects in *trans*. Because this study is one of the first human studies to publish on both *cis* and *trans* pQTLs, there may truly be more *trans* than *cis* variants affecting the proteome, or this observation could be biased because of our targeted quantification of human transcription factors and disease-related signaling proteins. The observation of an enrichment of *trans*, rather than *cis*, regulatory variants in complex human diseases such as type 2 diabetes and glucose homeostasis traits has supported the notion that many weak *trans* effects can influence mRNA (and putatively protein) levels and contribute to phenotypic variability.^54^ However, we appreciate that the shallower read depths of the RNA-sequencing data set (∼11 million mapped reads/individual) could have contributed to reduced power to detect eQTLs across this population and result in an underestimate in the proportion of true eQTLs present.

To assess pQTL reproducibility, we compared our results with those from a recent pQTL study that consistently quantified 2,279 proteins by isobaric tandem mass tag-based quantitative mass spectrometry across LCLs derived from 74 unrelated individuals from four populations in the HapMap Consortium.^14^ None of the 13 pQTLs identified in the YRI population from the Wu et al.^14^ study overlapped proteins quantified in our study, and two of the four pQTLs identified in our study at an FDR < 0.05 for proteins overlapping between both studies were in a concordant effect direction in the Wu et al. study. However, because of the small overlap of proteins (n = 61) and samples (n = 22) between studies, we do not feel that these conclusions are sufficient to make any larger inferences about pQTL reproducibility between studies. The fundamental differences between our approach and that of Wu et al.^14^ are in the methodology (mass spectrometry versus targeted antibody-based methods); the proteins chosen for measurement (highly abundant proteins detected by the mass spectrometer versus a smaller subset of targeted transcription factors and disease-related signaling molecules for which we had high-quality antibodies); and the population (68 YRI individuals versus a collection of four different ethnic panels with 1–53 individuals in each). However, 12% of *cis* and *trans* pQTLs identified in LCLs in this study at p < 10^−6^ replicate (here defined as concordant effect direction and nominal significance) in an unrelated cohort of 129 human cerebellum samples of European ancestry (data not shown), suggesting that many of these pQTLs are indeed real, tissue-independent associations (versus the null expectation of 2.5% replication).

We replicated a *trans* pQTL between rs60664312 and DIDO1 in an additional cohort of unrelated YRI LCLs and determined that this association was also a *cis* pQTL for the tRNA synthetase KARS in the same direction as DIDO1. We then performed siRNA knockdown on KARS and observed a concomitant reduction in DIDO1 protein levels for multiple cell lines. These data suggested that the *trans* pQTL for DIDO1 was a strong, reproducible association with a mechanism of action that involved alteration of KARS protein activity. *KARS* encodes Lysyl-tRNA synthetase (LysRS), which was originally described to catalyze the aminoacylation of lysyl-tRNAs in the cytoplasm and mitochondria.^55^ We hypothesize that RefSeq NP_001123561.1 (p.Thr623Ser) in LysRS could potentially affect its ability to be phosphorylated and subsequently its efficiency to aminoacylate lysyl-tRNAs. Altered KARS protein levels or function could result in concomitant altered abundances of downstream proteins such as DIDO1 that contain codons for this tRNA.

We identified many pQTLs that overlapped SNPs associated with complex traits and diseases, supporting previous mechanistic relationships and providing testable hypotheses about functional relationships that require further investigation. For example, we identified an intronic pQTL (rs1177283) associated with increased interferon regulatory factor 5 (IRF5) levels that was previously associated with increased risk of ulcerative colitis^56^ (MIM 266600) and celiac disease^57^ (MIM 212750). IRF5 is known to regulate type I interferon response and has been causally linked to autoimmune disease through variants driving elevated expression of multiple unique IRF5 isoforms.^58,59^ rs2738459 was previously associated with LDL cholesterol levels in a population of European descent^60^ and was associated in our study with ZNF207, a relatively uncharacterized zinc finger protein. We also identified many pQTLs that affected not only relative protein levels, but also the relative protein phosphorylation states, many of which overlapped with disease-associated loci. rs16852086 was associated with RPS6 (S240/244) protein phosphorylation levels in this study and previously with risk for chronic kidney disease (CKD) in a population of 67,093 Europeans,^61^ consistent with previous reports of altered basal RPS6 phosphorylation in CKD-induced rats.^62^ pQTLs offer the possibility that causal variants associated with complex diseases manifest their effects, at least in part, by altering protein levels. We suggest that pQTL analyses may be helpful for gaining additional biological insight into multidimensional phenotypes that is separate from that seen when performing eQTL analyses.

Lastly, we have provided a robust and scalable method for annotating human genetic variation that regulates the proteome. We demonstrate that meaningful information can be gained by a population-level assessment of the proteome along with the transcriptome. Although we examined only a subset of the proteome in this study, our focus on transcription factors will be of great utility for understanding genetic components of gene expression regulation by integrating ENCODE TF binding data, and our approach has no inherent limitation on the numbers of proteins or individuals that can be examined. Extending our approach to additional populations, cell types, and tissues will facilitate the identification of regulatory variation in complex traits and diseases. Incorporating this protein-omic data set with other “omic” data sets will provide a clearer understanding of the links between complex human traits and diseases with proteins and provide additional insight into global mechanisms of gene regulation.

## Figures and Tables

**Table 1:** Summary of Results for Association Analyses of Protein Levels

**SNP**	**Protein**	**Type**	**p Value**	**R**
rs60664312	DIDO1	*trans*	5.55 × 10^−17^	0.65
chr10.18096250	ZNF529	*trans*	4.21 × 10^−11^	0.45
rs751473	p-Raf(S338)	*trans*	8.91 × 10^−11^	0.45
rs7232517	ZNF645	*trans*	9.94 × 10^−11^	−0.44
rs72918427	ZNF414	*trans*	1.05 × 10^−10^	−0.44
rs145614393	ZMYND11	*trans*	2.80 × 10^−10^	−0.58
rs4691394	STAT3B	*trans*	5.66 × 10^−10^	−0.42
rs17020269	RUNX1	*trans*	6.72 × 10^−10^	0.52
rs141517138	OVOL1	*trans*	7.31 × 10^−10^	0.50
rs2016050	p-PDK1(S241)	*trans*	7.44 × 10^−10^	0.44
rs3893175	EP300	*trans*	7.85 × 10^−10^	0.50
rs11663180	GATA4	*trans*	1.16 × 10^−9^	−0.58
rs7331659	p-GAB1(Y659)	*trans*	1.18 × 10^−9^	0.42
rs16911722	TFAP2	*trans*	1.77 × 10^−9^	−0.45
rs4490893	IRF4	*trans*	2.28 × 10^−9^	−0.41
rs16870965	TFAP2	*cis*	2.72 × 10^−8^	−0.41
rs1638320	MED16	*cis*	1.22 × 10^−7^	0.44
rs10864374	ENO1	*cis*	1.22 × 10^−7^	0.40
rs7256500	ZNF266.75-100	*cis*	1.68 × 10^−7^	0.37

**Table 2:** Selected Overlap between pQTLs and GWAS SNPs

**Trait**	**SNP**	**PMID**	**Chr**	**Protein**	**p Value**
Conduct disorder (case status)	rs2184898	20585324	10	PRDM2	1.44 × 10^−8^
LDL cholesterol	rs2738459	20864672	19	ZNF207	1.32 × 10^−7^
Metabolic syndrome (bivariate traits)	rs320	21386085	8	ZMYND11	4.88 × 10^−7^
Platelet aggregation	rs1659838	20526338	10	ZMYND11	5.55 × 10^−7^
HIV-1 viral setpoint	rs6997496	22174851	8	FAK	1.15 × 10^−6^
Leprosy	rs10792430	22019778	11	OSR1	1.37 × 10^−6^
Primary biliary cirrhosis	rs10792430	21399635	11	OSR1	1.37 × 10^−6^
Blood pressure	rs17417407	21909110	10	TWIST1	1.39 × 10^−6^
Response to interferon beta therapy	rs9272105	21502966	6	SRC	1.44 × 10^−6^
Cytomegalovirus antibody response	rs931547	21993531	1	NCKAP1L	2.24 × 10^−6^
LDL cholesterol	rs4971544	21059979	2	NKX3-2	2.33 × 10^−6^
Cytomegalovirus antibody response	rs211228	21993531	6	MYST4	2.80 × 10^−6^

## Supplemental Data

Document S1. Figures S1–S8 and Table S1

Table S2. Antibody Screen Results

Table S3. Figure S4 Spot IDs

Table S4. Figure S5 Spot IDs

Table S5. Protein Quantifications

Table S6. siRNA Knockdown Results

Table S7. Significant Protein-Covariate Associations

Table S8. Significant RNA-Seq Covariate Associations

Table S9. pQTLs Identified at FDR = 0.20

Table S10. pQTL Validation Cohort Protein Quantifications

Table S11. eQTL and pQTL Genome Annotation Enrichments

Table S12. GWAS pQTL Overlap

Document S2. Article plus Supplemental Data

## Web Resources

The URLs for data presented herein are as follows:

1000 Genomes, http://browser.1000genomes.org
BIMBAM, http://stephenslab.uchicago.edu/software.html#bimbam
CRAN – Package lme4, http://cran.r-project.org/web/packages/lme4/index.html
CRAN – Package LMERConvenienceFunctions, http://cran.r-project.org/web/packages/LMERConvenienceFunctions/index.html
dbSNP, http://www.ncbi.nlm.nih.gov/projects/SNP/
eqtl.uchicago.edu, http://eqtl.uchicago.edu/cgi-bin/gbrowse/eqtl
Gene Expression Omnibus (GEO), http://www.ncbi.nlm.nih.gov/geo/
GWAS Catalog, http://www.genome.gov/gwastudies/
International HapMap Project, http://hapmap.ncbi.nlm.nih.gov/
Online Mendelian Inheritance in Man (OMIM), http://www.omim.org/
Python, https://www.python.org/
R statistical software, http://www.r-project.org/
RefSeq, http://www.ncbi.nlm.nih.gov/RefSeq
SeattleSeq Annotation 138 (129 used in this study), http://snp.gs.washington.edu/SeattleSeqAnnotation138/
UCSC Genome Browser, http://genome.ucsc.edu
