# Supplementary material for: Identification and Validation of Genetic Variants that Influence Transcription Factor and Cell Signaling Protein Levels
Source: Am J Hum Genet. 2014 Aug 7;95(2):194–208. doi: 10.1016/j.ajhg.2014.07.005 (PMC4129400; doi:10.1016/j.ajhg.2014.07.005)
Supplement: Document S1. Figures S1–S8 and Table S1 [file mmc1.pdf]

The American Journal of Human Genetics, Volume *95*
Supplemental Data

Identification and Validation of Genetic Variants that Influence Transcription Factor and Cell Signaling Protein Levels

Ronald J. Hause, Jr., Amy L. Stark, Nirav N. Antao, Lidija K. Gorsic, Sophie H. Chung, Christopher D. Brown, Shan S. Wong, Daniel F. Gill, Jamie L. Myers, Lida Anita To, Kevin P. White, M. Eileen Dolan, and Richard Baker Jones

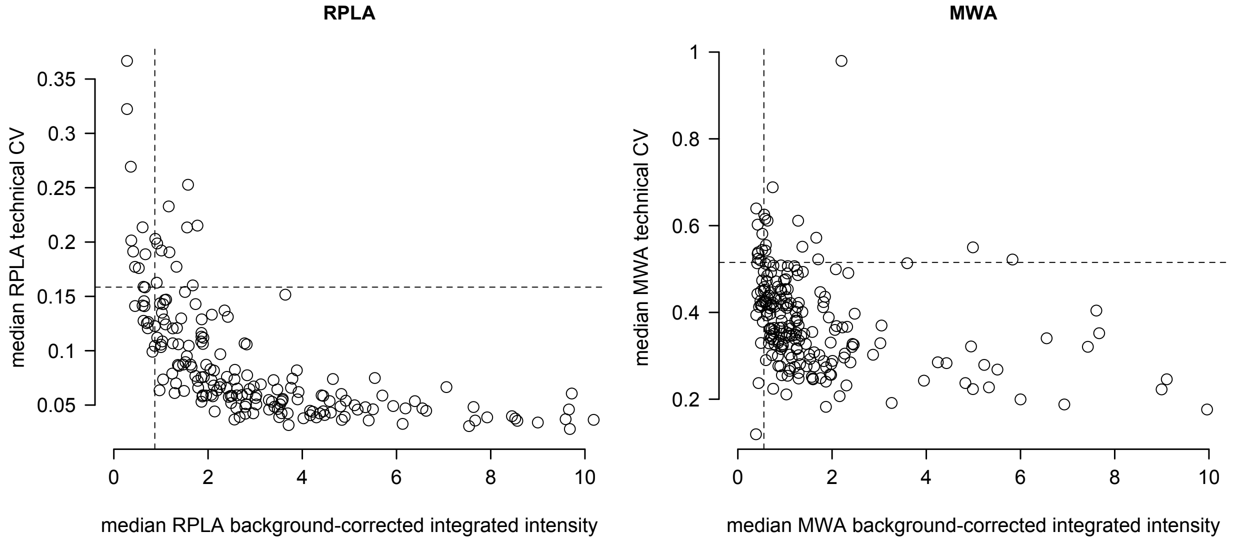

Figure S1: Technical variation plotted against antibody quality. Scatter plots of median background-corrected integrated intensities and median signal-to-noise ratios across all samples versus the median technical coefficient of variation for each antibody. Lines designate the filtering thresholds we used for selecting high-confidence protein quantifications.

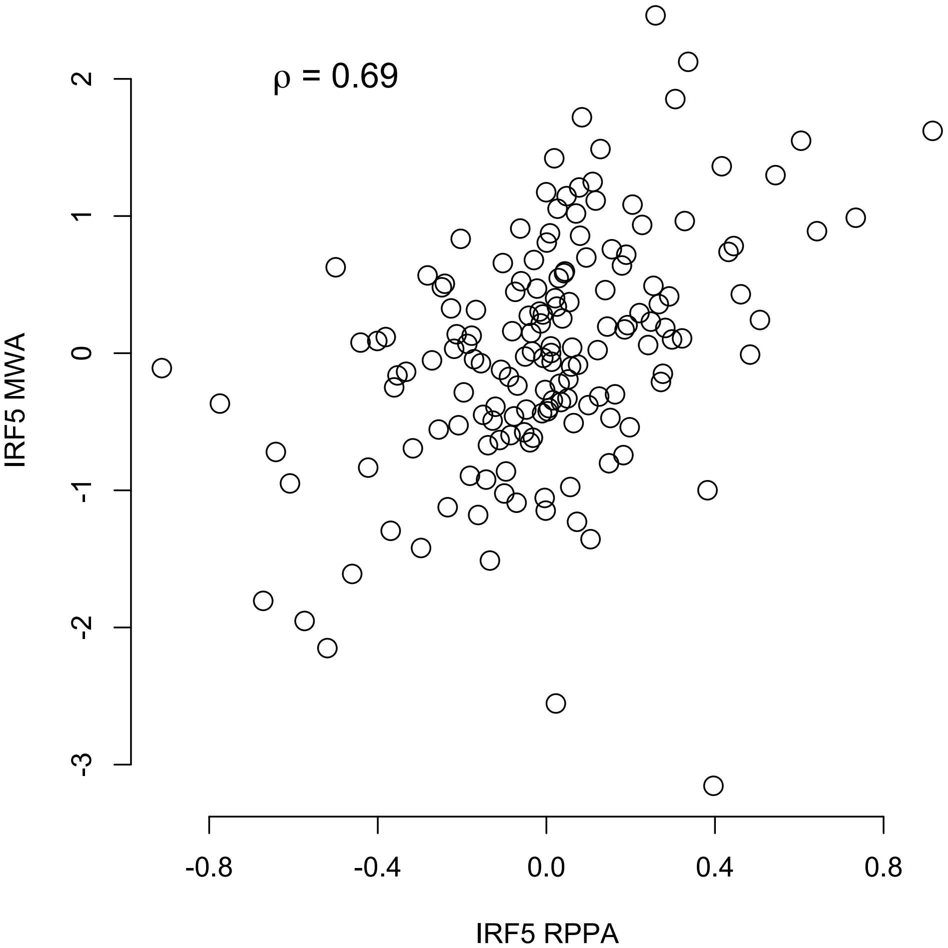

Figure S2: Inter-platform correlation. Correlation between the relative protein levels quantified for the same antibodies across microwesterns (x-axis) versus RPLAs (y-axis) for all 204 samples for an antibody directed against IRF5.

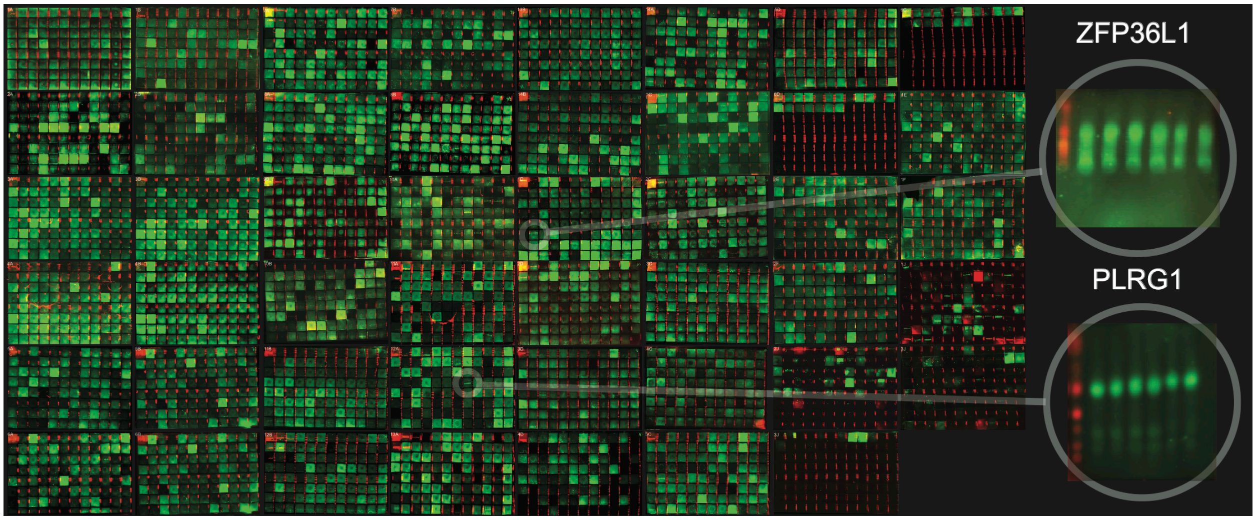

Figure S3: Sample results from screening 4,366 antibodies across six pooled samples using 96-well microwestern arrays. Images are shown for one representative blot at a single scan intensity from each plate of screened antibodies at 1:1000 dilutions. ZNFP36L1 passed the screen for subsequent population-level quantification by MWAs, whereas PLRG1 passed the screen for subsequent population-level RPLA quantification.

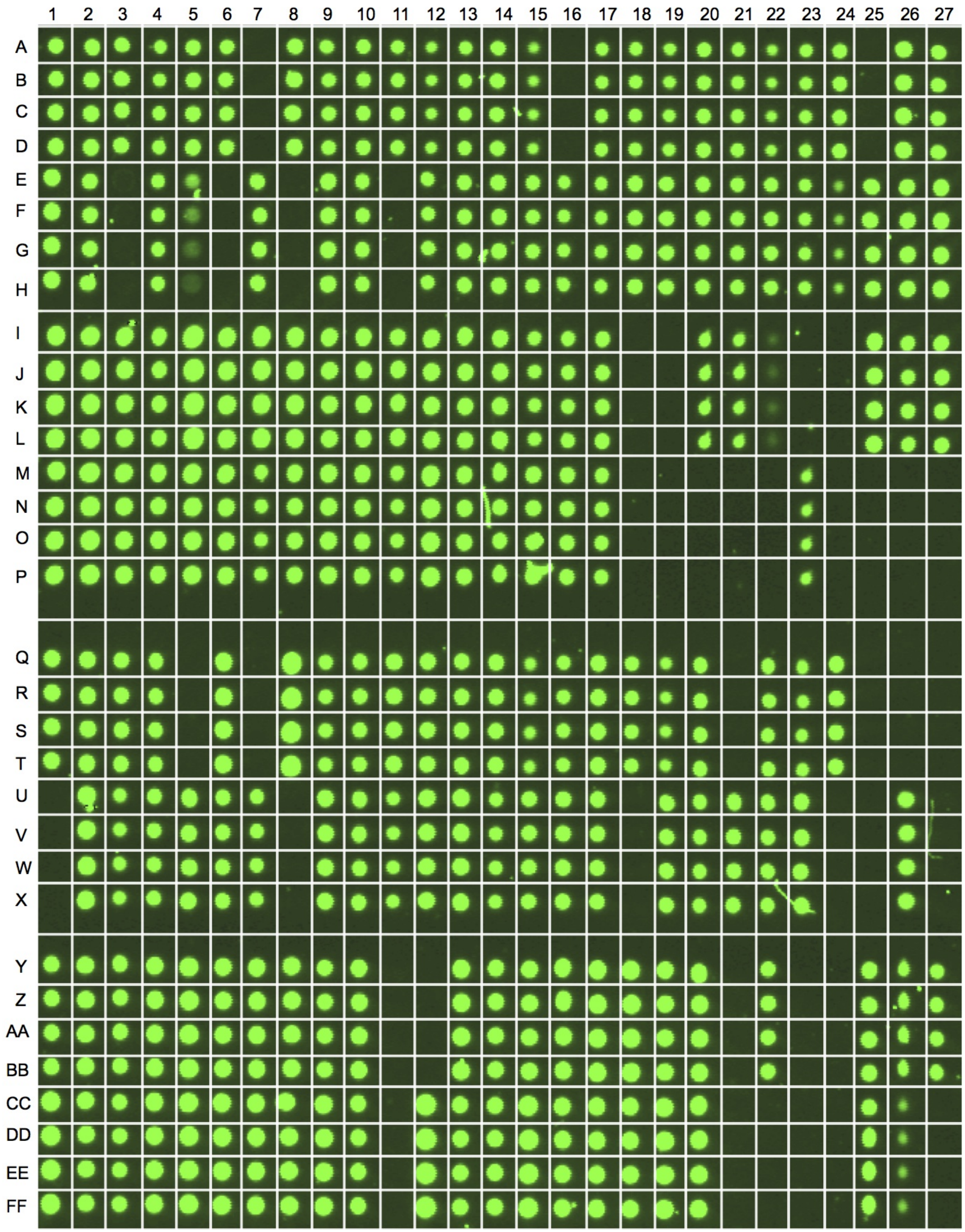

Figure S4: Sample population-level quantification of DIDO1 (Antibody ID #440) by reverse phase protein arrays. Sample IDs can be found in Supplementary Table 2.

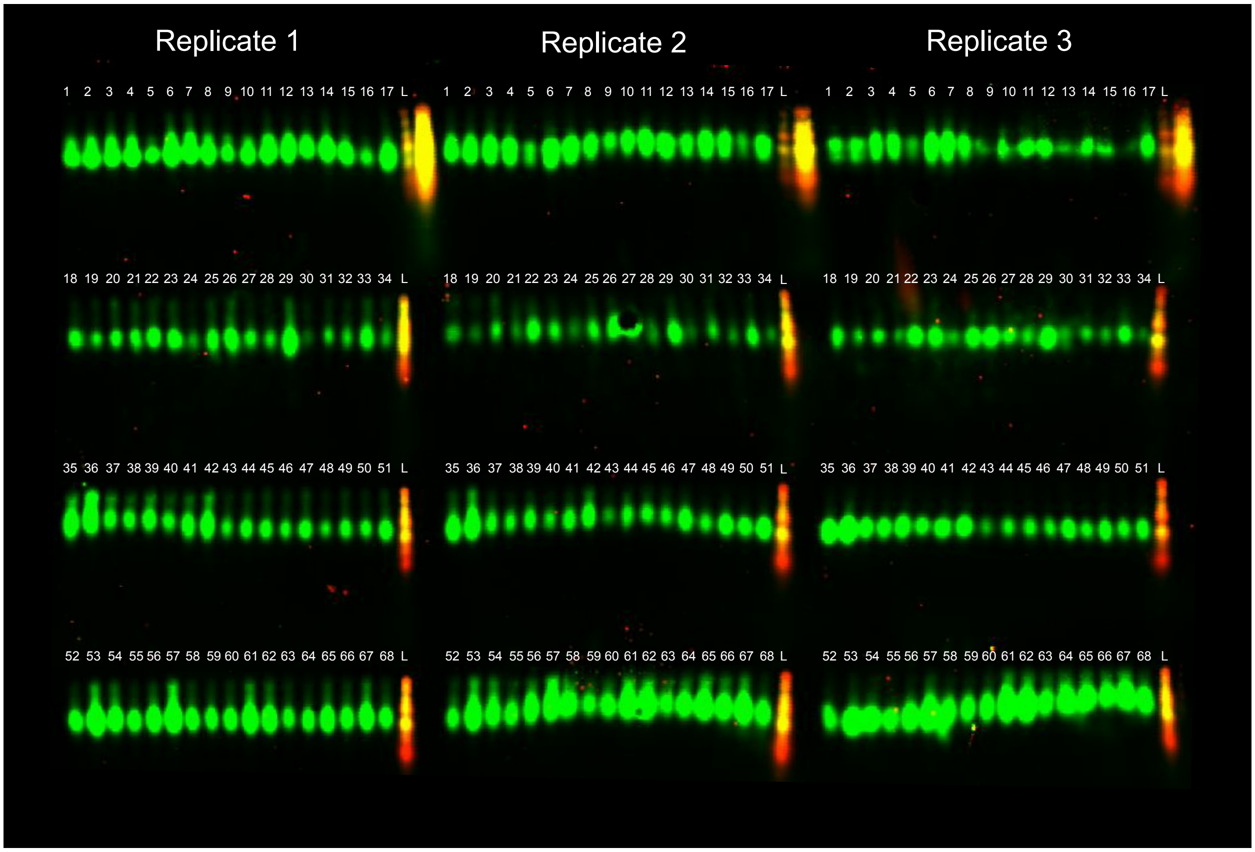

Figure S5: Sample population-level quantification of Src (Antibody ID# 208) by quadrant microwestern arrays. Sample IDs can be found in Supplementary Table 3.

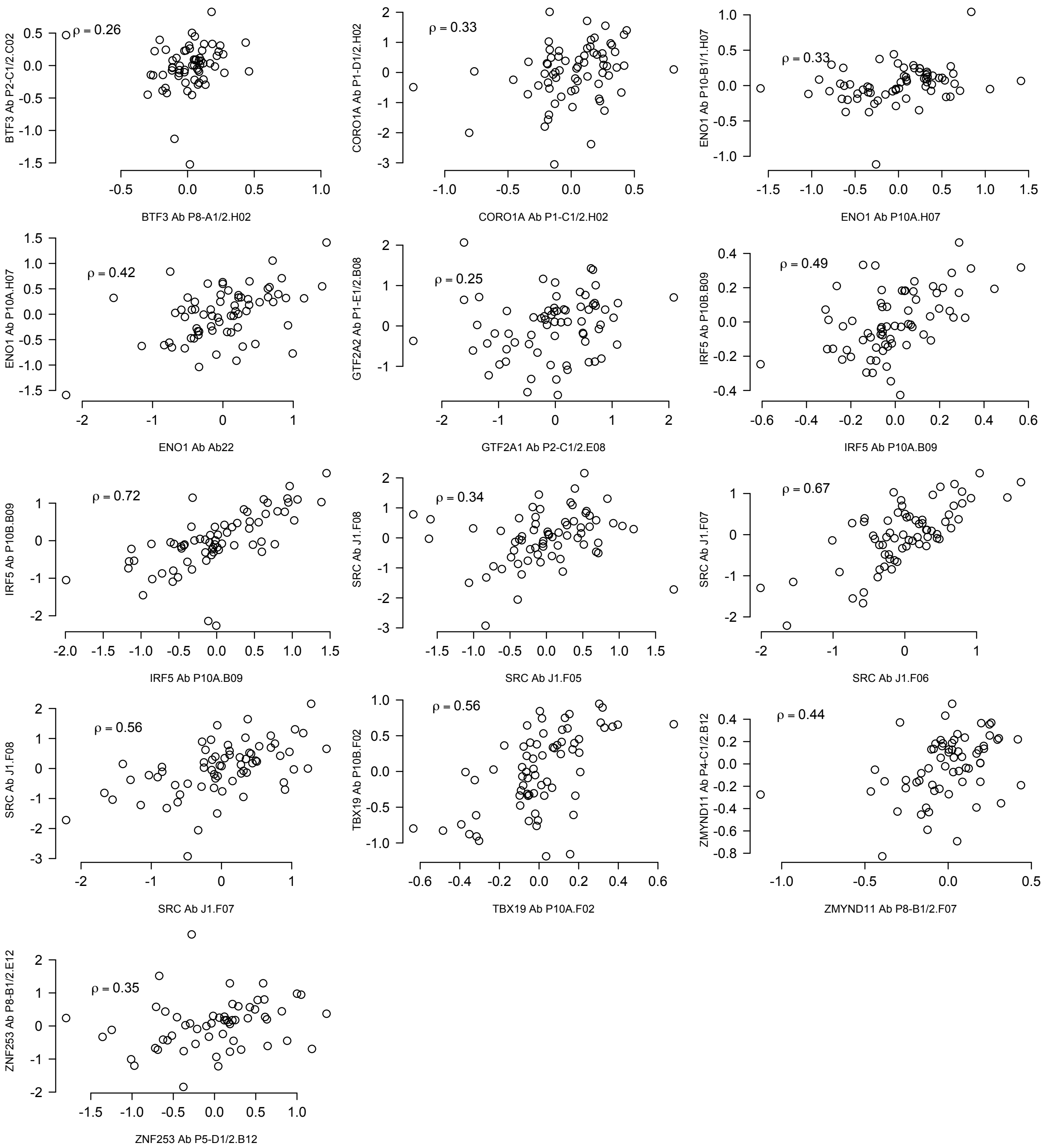

Figure S6: Inter-antibody correlations. Correlations between the relative inter-individual protein levels quantified by microwesterns with two distinct antibodies directed against thirteen separate proteins.

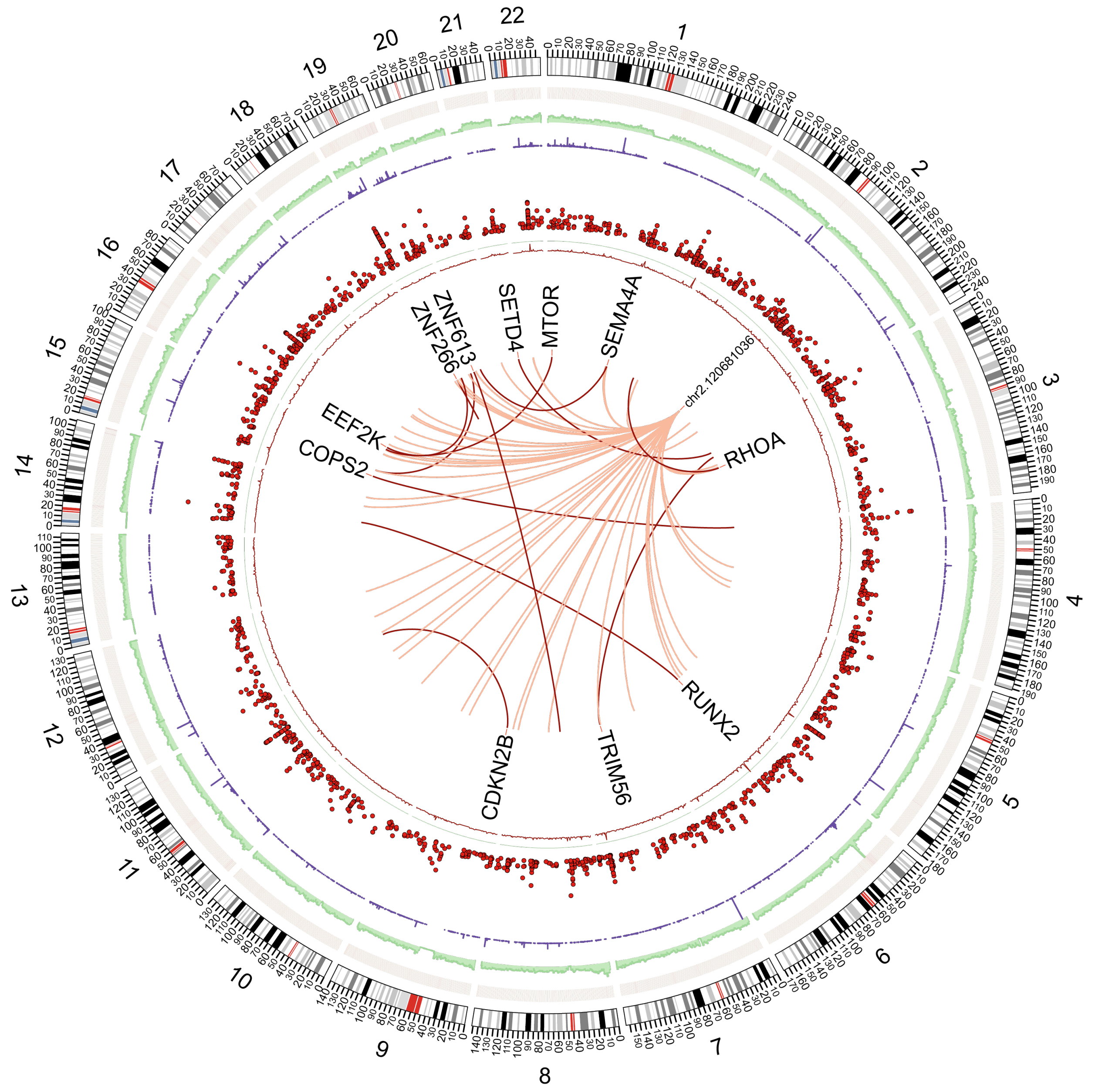

Figure S7: Genetic variants affecting mRNA levels globally and in *cis*. Circos plot for the eQTL association results for the subset of genes that were also quantified at the protein level. The rims, in order from outside inward, depict (1) a Manhattan plot of the -$\log_{10}(P)$ for each eQTL identified at $P < 10^{-4}$) (for plotting clarity) and (2) the top 12 (constrained due to space) significant eQTLs ($P < 10^{-10}$) and the top master regulatory SNP for each dataset, and (3) the innermost network depicts spokes between QTLs and their regulated genes, with light red spokes depicting the top QTL interactions and dark red spokes depicting proteins associated with the top master regulatory SNP for each dataset. Such SNPs include chr2.120681036, which marks an mRNA-specific master regulatory region.

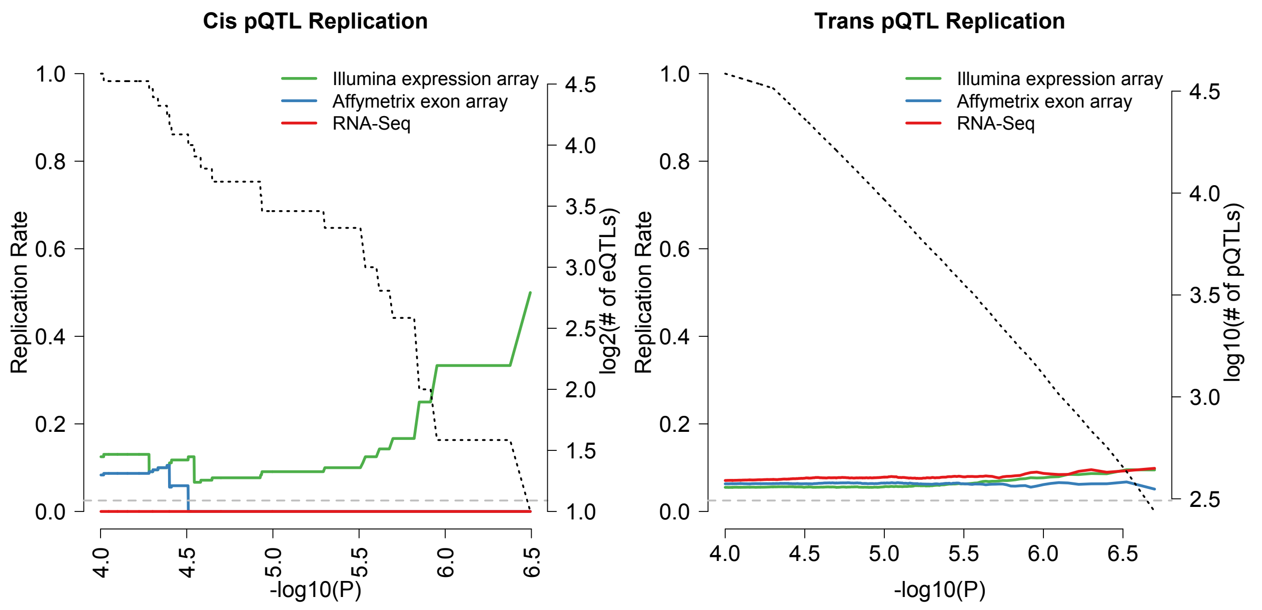

Figure S8: pQTL evidence at the mRNA level. a. *cis*- and **b.** *trans*-pQTL replication rate (y-axis) is depicted as a function of the *P*-value in the protein data (x-axis) for Illumina expression array (green), Affymetrix exon array (blue) and transcriptome sequencing (red). The black dashed lines denote the number of eQTLs at each discovery *P*-value. The grey dashed line denotes the null expectation of replication rate at $P < 0.05$ with concordant effect direction.

Supplementary Table S1 - siRNA lists

FlexiTube GeneSolution GS11083 for DIDO1
Entrez gene ID: 11083 (4 siRNAs, 1 nmol each)
SI03080042
SI03111185
SI00132552
SI00132559

FlexiTube GeneSolution GS10025 for MED16
Entrez gene ID: 10025 (4 siRNAs, 1 nmol each)
SI02635346
SI03057103
SI00077854
SI00077868

FlexiTube GeneSolution GS3235 for HOXD9
Entrez gene ID: 3235 (4 siRNAs, 1 nmol each)
SI04249028
SI04907700
SI04157846
SI04205873

FlexiTube GeneSolution GS7633 for ZNF79
Entrez gene ID: 7633 (4 siRNAs, 1 nmol each)
SI03230836
SI04339748
SI03140879
SI03170300

FlexiTube GeneSolution GS221785 for ZNF498
Entrez gene ID: 221785 (4 siRNAs, 1 nmol each)
SI04324089
SI04360923
SI03124303
SI04223583

FlexiTube GeneSolution GS3196 for TLX2
Entrez gene ID: 3196 (4 siRNAs, 1 nmol each)
SI00745836
SI04370730
SI00745815
SI00745822

FlexiTube GeneSolution GS11151 for CORO1A
Entrez gene ID: 11151 (4 siRNAs, 1 nmol each)
SI03231753
SI04364948
SI00351848
SI03188507

FlexiTube GeneSolution GS7287 for TULP1
Entrez gene ID: 7287 (4 siRNAs, 1 nmol each)
SI00050764
SI03067750
SI00050750
SI00050757

FlexiTube GeneSolution for ATF2
Entrez gene ID: 1386 (4 siRNAs, 1 nmol each)
SI02780309
SI00305872
SI03095946
SI05037508

FlexiTube GeneSolution GS148266 for ZNF569
Entrez gene ID: 148266 (4 siRNAs, 1 nmol each)
SI04219628
SI04372963
SI03156503
SI04202639

FlexiTube GeneSolution GS8243 for SMC1A
Entrez gene ID: 8243 (4 siRNAs, 1 nmol each)
SI02655219 (Experimentally verified)
SI00087241
SI00087255
SI00087248

FlexiTube GeneSolution GS6194 for RPS6
Entrez gene ID: 6194 (4 siRNAs, 1 nmol each)
SI04339860
SI04342821
SI00708008
SI04219152

FlexiTube GeneSolution GS3071 for NCKAP1L
Entrez gene ID: 3071 (4 siRNAs, 1 nmol each)
SI04298112
SI04316851

SI00435568
SI04235602

FlexiTube GeneSolution STAT3
1. SI02662338
2. SI00048377
3. SI02662898
4. SI00048384
5. SI00048363
6. SI00048370

Flexitube GeneSolution GS3735 for KARS
Entrez gene ID: 3735 (4 siRNAs, 1 nmol each)
SI00450422
SI03241588
SI04212075
SI04262069
